# Transcriptome Analysis of Ice Plant Growth-Promoting Endophytic Bacterium *Halomonas* sp. Strain MC1 to Identify the Genes Involved in Salt Tolerance

**DOI:** 10.3390/microorganisms8010088

**Published:** 2020-01-09

**Authors:** Jian Zhang, Pengcheng Wang, Hongmei Tian, Zhen Tao, Tingting Guo

**Affiliations:** 1 Institute of Horticulture, Anhui Academy of Agricultural Sciences, Hefei 230031, Anhui, Chinapriorpost06@aliyun.com (Z.T.);; 2 Key Laboratory of Genetic Improvement and Ecophysiology of Horticultural Crops, Hefei 230031, Anhui, China; 3 School of Life Sciences, Anhui Agricultural University, Hefei 230036, Anhui, China

**Keywords:** bacterial growth, endophytes, *Halomonas* sp., salt stress, transcriptome analysis

## Abstract

Salt stress is an important adverse condition encountered during plant and microbe growth in terrestrial soil ecosystems. Currently, how ice plant (*Mesembryanthemum crystallinum*) growth-promoting endophytic bacteria (EB) cope with salt stress and regulate growth and the genes responsible for salt tolerance remain unknown. We applied RNA-Seq technology to determine the growth mechanism of the EB *Halomonas* sp. MC1 strain and the genes involved in salt tolerance. A total of 893 genes were significantly regulated after salt treatment. These genes included 401 upregulated and 492 downregulated genes. Gene Ontology enrichment and Kyoto Encyclopedia of Genes and Genomes analysis revealed that the most enriched genes included those related to the outer membrane-bounded periplasmic space, ATPase activity, catabolic process, and proton transmembrane transport. The quantitative real-time polymerase chain reaction data were similar to those obtained from RNA-Seq. The MC1 strain maintained survival under salt stress by regulating cellular and metabolic processes and pyruvate metabolism pathways such as organic and carboxylic acid catabolic pathways. We highlighted the response mechanism of *Halomonas* sp. MC1 to fully understand the dynamics of complex salt–microbe interactions.

## 1. Introduction

Abiotic environmental factors considerably affect microbial growth and play a vital role in plant growth and productivity [1]. The salinity of arable lands under field and greenhouse conditions has become a major problem in agricultural production [2]. Salinity has become a major environmental stress factor because it not only limits plant growth, but also microbial survival [3]. Various strategies have been developed to decrease the toxic effects of salt on plant or bacteria growth. At present, plant growth-promoting bacterial endophytes colonize host plant tissues without causing disease symptoms by interacting with host plants, enhancing salt stress tolerance [4]. Plants form a symbiotic relationship with endophytic bacteria (EB) throughout their growth and development. This relationship benefits plants in terms of salt stress damage or pathogen invasion as well as sustained plant growth in harsh environments [5]. For instance, inoculating crop seeds and seedlings with EB is an unconventional approach to alleviate salt stress, and thus supports plant growth [6,7,8]. Although the beneficial effects of EB on salinized plants have been well studied, most of the underlying physiological and molecular mechanisms and gene expression levels need to be identified to optimize the applications of EB in agricultural production.

Beneficial EB colonize the rhizosphere/endorhizosphere of plants and promote growth; they have been discovered in many plant species and can be isolated from sorghum, soybean, and native perennial plants [9], suggesting that EB can be a promising alternative to alleviate the toxic effects of salinity. However, entry for endophytes may occur in various plant regions, especially the root zone [10,11,12,13]. Thus, beneficial EB are potential resources for reducing the negative effects of salinization. Many studies have focused on the species of EB including *Bacillus*, *Enterobacter*, *Klebsiella*, *Pseudomonas*, and *Xanthomonas* [9]. The recent availability of the genome sequences of important EB has provided new insights into the molecular pathways involved in plant–endophyte symbiosis [14] and led to a new understanding of the adaptation of EB to salt stress [15,16,17]. The analysis of novel gene responses of EB may provide a potential method to cope with salinity. EB can enhance *Arabidopsis thaliana* growth under salt stress and can tolerate high salt concentrations and show a potential competitive advantage of surviving in saline soils [18,19]. The complete genome sequence of the endophyte *Bacillus flexus* helps in revealing the plant growth-promoting mechanism of EB [20]. However, limited studies have focused on the EB genes involved in salt stress response. A mechanistic understanding of how EB improves plant growth at morphological, growth, and molecular levels is vital for EB application [21,22,23] and could help in identifying useful EB that can protect plants against salt stress.

Ice plants (*Mesembryanthemum crystallinum*) display remarkable tolerance to diverse abiotic stresses, especially salt stress, and they are consumed as vegetables [24,25]. Various EB were obtained from this plant in our previous work. In this study, we selected MC1, a *Halomonas* sp. strain that has been identified as an endophytic nitrogen-fixing bacterium. This strain exhibits NaCl tolerance and plant growth-promoting traits [26]. However, the molecular mechanism of MC1’s response to salt stress at the genetic level remains unclear. Therefore, the current study investigated the transcriptomes of *Halomonas* sp. MC1 under salt stress through high-throughput sequencing to elucidate the genes involved in salt tolerance. Notably, we expanded the current knowledge by providing new information on the bacteria involved in salt resistance.

## 2. Materials and Methods

### 2.1. Strain and Culture Condition

*Halomonas* sp. strain MC1 (GenBank accession number: MF431770; Transcriptome number: SRP235179) was isolated from the interior stem of ice plants and verified as a bacterial endophyte. The strain shows plant growth-promoting effects and improved growth of cabbage seedlings under salt stress [26]. MC1 was cultivated on nutrient solid agar plates (5.0 g/L yeast extract, 10.0 g/L peptone, and 15.0 g agar at pH 6.9–7.1) containing 1.71 M NaCl at 25 °C ± 2 °C for five days. Fresh cells were stored with 40% glycerol at −80°C until further analysis.

### 2.2. Induction of Salt Tolerance and Bacterial Collection

The salt-resistance activity of MC1 was determined in an activated bacterial culture grown in solid medium with (1.71 M NaCl) or without (CK, 0 M NaCl) salt. The concentration of NaCl was determined based on the optimal growth condition of strain MC1 [26]. Bacterial concentration was adjusted to 10^8^ CFU/mL. The bacteria were grown in two kinds of cultures for three days at 25 °C, and the bacterial growth cultures were collected. The cultured bacteria were transferred to test tubes, frozen with liquid nitrogen, and stored at −80 °C until further analyses. All treatments were performed in triplicate.

### 2.3. RNA Extraction, Library Construction, and Sequencing

Total RNA was extracted from the bacterial cultures by using TRIzol^®^ Reagent (Invitrogen) in accordance with the manufacturer’s instructions. Genomic DNA was removed using DNase I (TaKara, Dalian, China). Then, RNA quality was determined with a NanoDrop 2000 spectrophotometer (Thermo Fisher Scientific, Waltham, MA, USA). A high-quality RNA sample (8 µg, optical density (OD)_260/280_ = 1.8–2.2; OD_260/230_ ≥ 2.0) was used to construct the sequencing library. All experimental and control treatments were performed with three biological replicates per group. The RNA was fragmented into small pieces and used as a template for cDNA synthesis according to the TruSeq RNA preparation kit from Illumina (San Diego, CA, USA). Apolymerase chain reaction (PCR) solution containing a mixture of dATP, dGTP, dCTP, and dUTP was used, and the reaction condition was set at 16 °C for 1 h. In brief, libraries were size-selected for cDNA target fragments of 200–300 bp on 2% low-range ultra-agarose, followed by PCR amplification using Phusion DNA polymerase (NEB). After quantification with TBS380, the paired-end libraries were sequenced by Shanghai Biozeron Biothchology Co. Ltd. (Shanghai, China) with an Illumina HiSeq PE 2 × 151 bp read length.

### 2.4. Quality Control and Mapping of Reads

The raw paired-end reads were subjected to quality control by using Trimmomatic with default parameters (Available online: http://www.usadellab.org/cms/uploads/supplementary/Trimmomatic). Then, the clean reads were separately aligned to the reference genome in orientation mode using Rockhopper (Available online: http://cs.wellesley.edu/~btjaden/Rockhopper/) software. We used the Rockhopper system for the computational analysis of bacterial RNA-Seq data due to its comprehensive and user-friendly properties [27]. Rockhopper uses RNA sequencing reads generated by high-throughput sequencing technology as the input. This software was used to calculate the gene expression levels under the default parameters. Raw reads such as those containing adapters, more than 10% unknown nucleotide content, and more than 50% low-quality base content were removed to obtain high-quality clean reads.

### 2.5. Transcriptome De Novo Assembly and Annotation

The clean reads were obtained and selected from the raw data by filtering out adaptors and low-quality (Q ≤ 20) reads by following criteria: shorter than 75 bp, N-base ratio over 10%, or mismatch/adapter sequences. De novo assembly was performed to generate transcriptsby using the trinity method [28]. The resulting unigenes were used for BLAST searches in databases such as the National Center for Biotechnology Information (NCBI) (Available online: http://www.ncbi.nlm.nih.gov/), Swissprot (Available online: http://www.expasy.ch/sprot/), and Kyoto Encyclopedia of Genes and Genomes (KEGG) (Available online: http://www.genome.jp/kegg/). Gene Ontology (GO) annotation was conducted with the Blast GO program to classify the unigenes [29]. All the unigenes were uploaded to the KEGG metabolic pathway database for pathway assignment [30,31].

### 2.6. Differential Expression Analysis and Functional Enrichment

The gene expression level was calculated using the fragments per kilobase of read per million mapped reads method to identify the differentially expressed genes (DEGs) between samples under two treatments. EdgeR (Available online: https://bioconductor.org/packages/release/bioc/html/edgeR.html) was used for differential expression analysis. The following criteria were selected to identify DEGs: (i) a logarithmic of fold change greater than 2 and (ii) a false discovery rate of less than 0.05. GO functional enrichment and KEGG pathway analyses were performed with Goatools (Available online: https://github.com/tanghaibao/Goatools) and KOBAS (Available online: http://kobas.cbi.pku.edu.cn/home.do), respectively, to understand the functions of the DEGs. The DEGs were considered significantly enriched in GO terms and metabolic pathways in accordance with the reported method under multiple test corrections [32] when their corrected *p*-value was less than 0.05 using Fisher’s test. In order to improve statistical significance, the *q*-value was used to estimate the false discovery rate (FDR). The MC1 nucleotide sequences of the 16S rDNA were subjected to BLAST analysis with the NCBI database (Available online: http://blast.ncbi.nlm.nih.gov/Blast.cgi), and sequences with high similarity scores were selected and downloaded. A phylogenetic tree of the MC1 strain was constructed using MEGA 6.0 by using neighbor-joining analysis. The gene circle of MC1 was mapped with Circos v0.64 (Available online: http://circos.ca/).

### 2.7. Quantitative Real-Time Polymerase Chain Reaction (qRT-PCR) Validation

Given the large number of DEGs in this work, six DEGs were selected for qRT-PCR analyses to determine whether the gene expressions were consistent with the RNA-Seq results. The primers used were designed with Primer 5.0 (Table S1). Total RNA was isolated using the Tiangen (Biotech Co. Ltd., Beijing, China) reagent in accordance with the manufacturer’s protocol, and RNA was utilized for cDNA synthesis using reverse transcriptase. Three technical replicates for each sample were conducted in qPCR runs. The 16S rRNA gene of *Halomonas* sp. was used as the control gene. The Fast Real-time PCR System (Applied Biosystems, Foster, CA, USA) was used to perform qRT-PCR.

### 2.8. Statistical Analysis

The data were collected, and mean analysis was conducted using EXCEL. The data were also analyzed via one-way analysis of variance (Fisher’s least significant difference (LSD) test), and the means were separated through Fisher’s protected LSD test using the SPSS package (version 19.0). Differences obtained at the *p* ≤ 0.05 level were considered significant.

## 3. Results

### 3.1. Data Processing and Analysis

**Figure 1:**
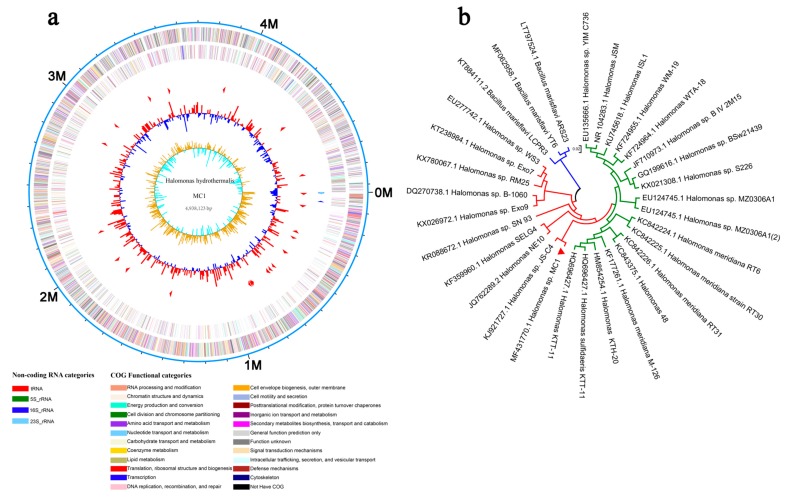
Genome-wide assessment. (**a**) Circular plot of reads mapped to the *Halomonas* sp. MC1 genome. (**b**) Phylogenetic tree constructed on the basis of 16S rDNA sequences of neighboring species using the neighbor-joining method. The bars represent 0.02 substitutions per nucleotide position.

The number of raw reads obtained in this experiment ranged from 13,471,728 to 16,857,140. The Q30 (percentage of reads with lengths exceeding 30 bp in the total bases) of MC1 exceeded 90%. The clean reads and bases ranged from 11,691,054 to 14,554,380 bp and from 1,726,157,998 to 2,094,489,027 bp, respectively. The mapped rate reached the maximum of 93.13% after filtering out the contaminated and low-quality sequences (Table 1). The Q30 of clean reads exceeded 93.00%. The error was less than 0.02%. The sequences were subsequently visualized using a genome browser (Figure 1a). The nucleotide sequences of the MC1 16S rDNA were subjected to BLAST analysis, and a phylogenetic tree of MC1 was constructed on the basis of the 16S rDNA sequences using neighbor-joining analysis (Figure 1b). This strain is determined as closely related to the genus *Halomonas* sp.

### 3.2. Number of DEGs

**Figure 2:**
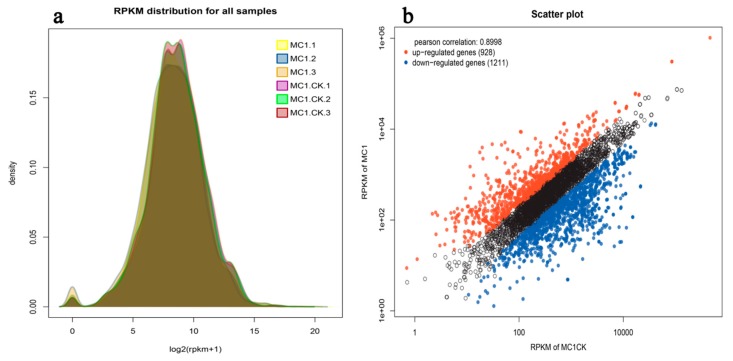
Expression level (reads per kilobase of transcript per million reads mappedscores) distribution map (**a**) and visualized scatter plot of differentially expressed genes (**b**). Black scatters represent undifferentially expressed genes.

As shown in Figure 2a, based on the values of the reads per kilobase of transcript per million reads mapped (RPKM), the measured peaks of gene expression in six biological samples were similar. However, the gene expressions of three salt-treated (1.71 M NaCl) samples (MC1.1, MC1.2, and MC1.3) were lower than those of CK (MC1.CK.1, MC1.CK.2, and MC1.CK.3, 0 M NaCl). The gene expression of CK was about 2.32% higher than that of MC1. As shown in Figure 2b, 928 genes were upregulated, and 1211 genes were downregulated. The Pearson correlation index was 0.8998, as inferred from the scatter plot. Salt stress treatment resulted in differences in expression between the two group samples.

**Figure 3:**
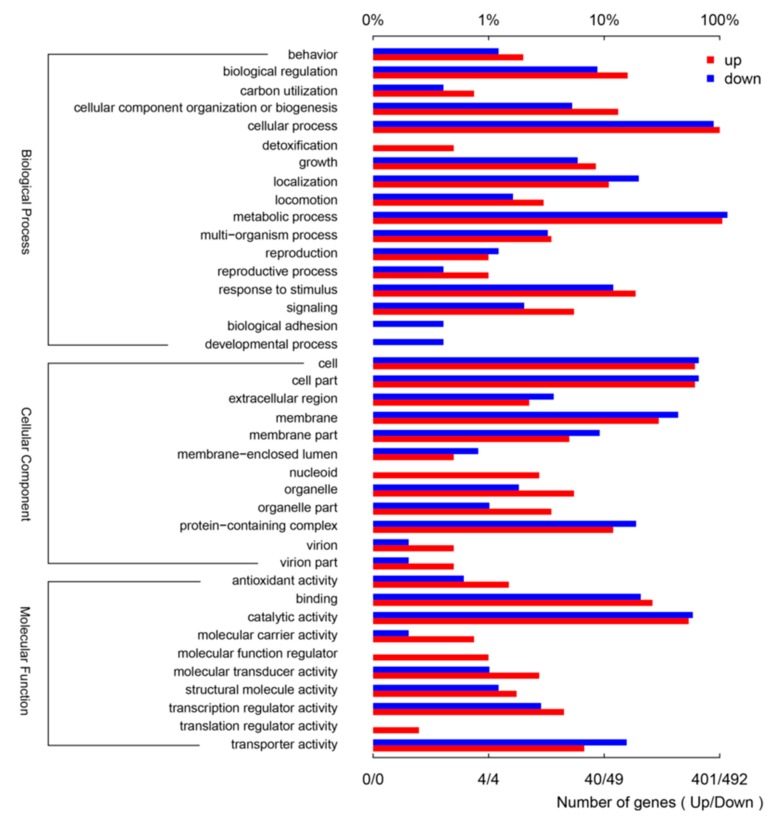
Chart summarizing the results of Gene Ontology enrichment analysis under salt treatment. Three categories, namely, biological processes, cellular components, and molecular functions, are shown. Up and down classification of differentially expressed genes are also marked.

Significant changes were observed in 893 genes including 401 upregulated and 492 downregulated ones after salt treatment (Figure 3). A total of 27 (1.26%) genes in the biological process component were annotated to the outer membrane-bounded periplasmic space and accounted for 0.87% of the total genes. In terms of molecular function, 110 genes were involved in ATPase activity coupled with the transmembrane movement of substances and accounted for 5.14% of the total genes. For biological process, 254 genes were annotated to the catabolic process and accounted for 11.87% of the total genes. The number of genes in ion transmembrane transport reached 64 and accounted for 2.99% of the total genes (Table 2).

### 3.3. GO Analysis of DEGs

**Figure 4:**
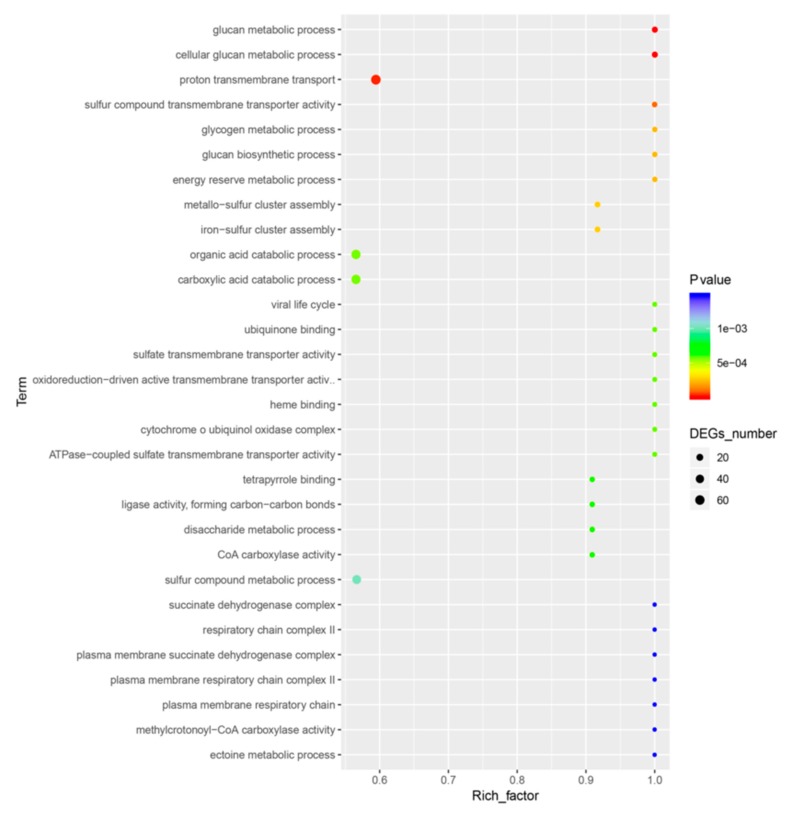
Top Gene Ontology enriched pathways. A large rich factor indicates a high degree of enrichment.

The differentially expressed unigenes of *Halomonas* sp. MC1 were subjected to functional analysis. Three domains (biological process, cellular components, and molecular functions) were used in the GO functional classification of genes. In this study, all the DEGs were annotated to the terms in the GO database. Our data showed that the majority of genes were annotated to cellular components, molecular functions, and biological processes. GO analysis revealed that 1.26%, 0.47%, and 0.46% of the identified genes were involved in the outer membrane-bounded periplasmic space (GO:0030288), plasma membrane proton-transporting ATP synthase complex (GO:0045260), andproton-transporting ATP synthase complex (GO:0045259), respectively. Of the 17 molecular functions identified, ATPase activity coupled with the transmembrane movement of substances (GO:0042626), active transmembrane transporter activity (GO:0022804), and primary active transmembrane transporter activity (GO:0015399) were significantly enriched with total gene proportions of 5.14%, 3.41%, and 3.03%, respectively. Of the 1000 biological processes identified, catabolic process (GO:0009056), proton transmembrane transport (GO:1902600), and ion transmembrane transport (GO:0034220) were significantly regulated and enriched with total gene proportions of 11.87%, 3.09%, and 2.99%, respectively (Figure 4, Table 2).

### 3.4. KEGG Analysis of DEGs

**Figure 5:**
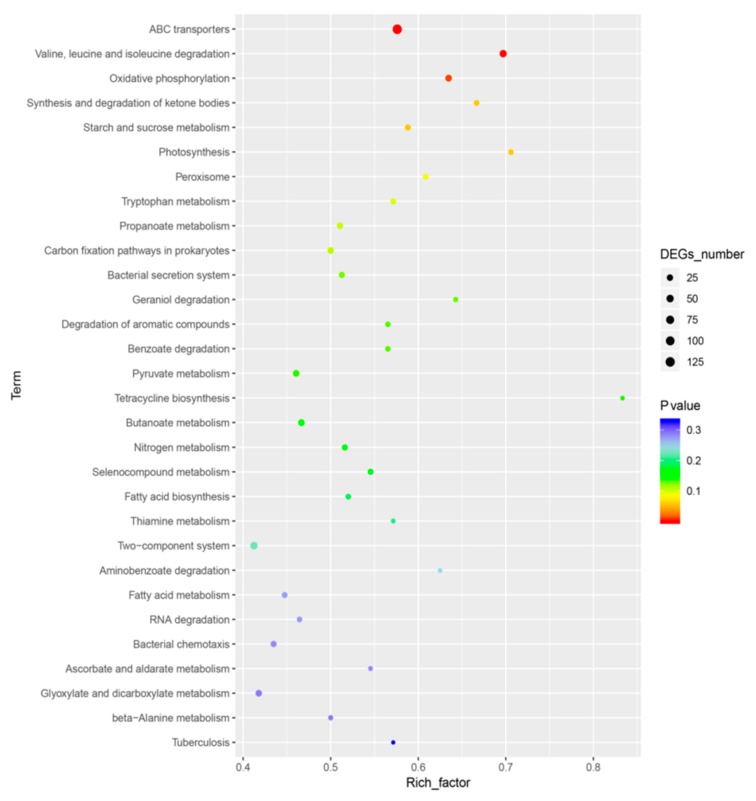
Top pathways enriched in the Kyoto Encyclopedia of Genes and Genomes analysis. A large rich factor indicates a high degree of enrichment.

The DEGs in the MC1 strain were matched to 104 different KEGG pathways.ABC transporters were the most common pathway. A total of 224 genes including 129 DEGs were designated as ABC transporters. A total of 66 genes including 46 DEGs participate in the valine, leucine, and isoleucine degradation pathway (Figure 5, Table 3).

### 3.5. Clustering Analysis of DEGs

**Figure 6:**
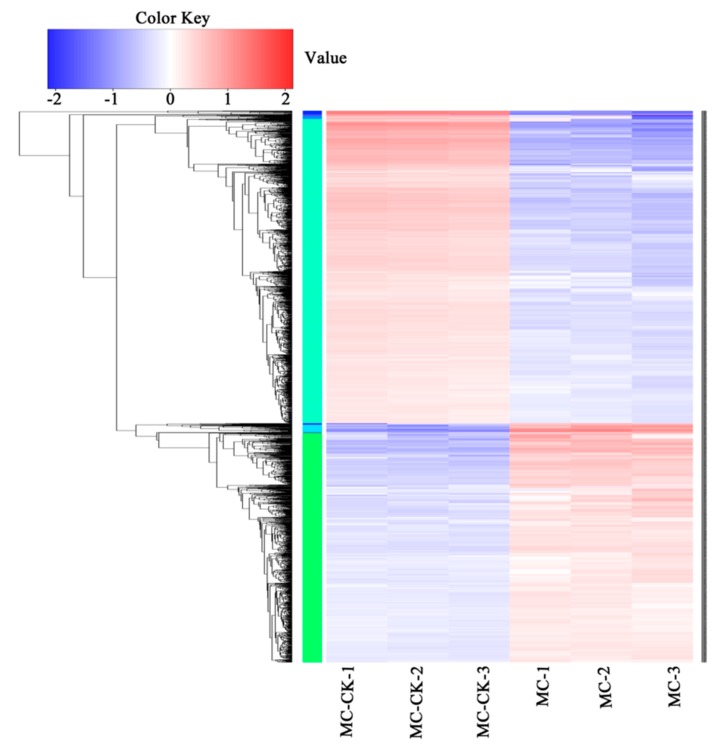
Clustering heatmap of differentially expressed genes.

**Figure 7:**
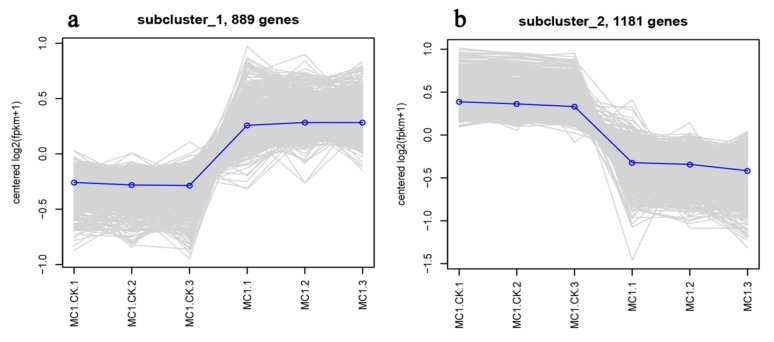
MC1 vs. MC1.CK differentially expressed genes sub-cluster trend lines. A total of 889 genes were analyzed by Branch 1 (**a**), and 1181 genes were analyzed by Branch 2 (**b**). The number of downregulated genes was higher than that of upregulated genes under high salinity.

Figure 6 shows the results of the DEG clustering analysis. The number of upregulated genes under salt-treated MC1 was lower than that of downregulated genes. Cluster analysis showed that the six samples were divided into two different sections (Figure 6). The genes with the most pronounced upregulation were related to cellular regulation of biological process such as MC1004145, MC1000224, and MC1000519, and those with the most pronounced downregulation comprised MC1005273, MC1004502, and MC1000699. Those selected genes were related to cell part process, which might be involved in signals interaction or cell formation under salt stress. In order to figure out the trend of each branch, a total of 889 and 1181 genes were analyzed by Branch 1 (Figure 7a) and Branch 2 (Figure 7b), respectively. The number of downregulated genes was larger than that of the upregulated genes under high-salinity treatment.

### 3.6. qRT-PCR Validation

**Figure 8:**
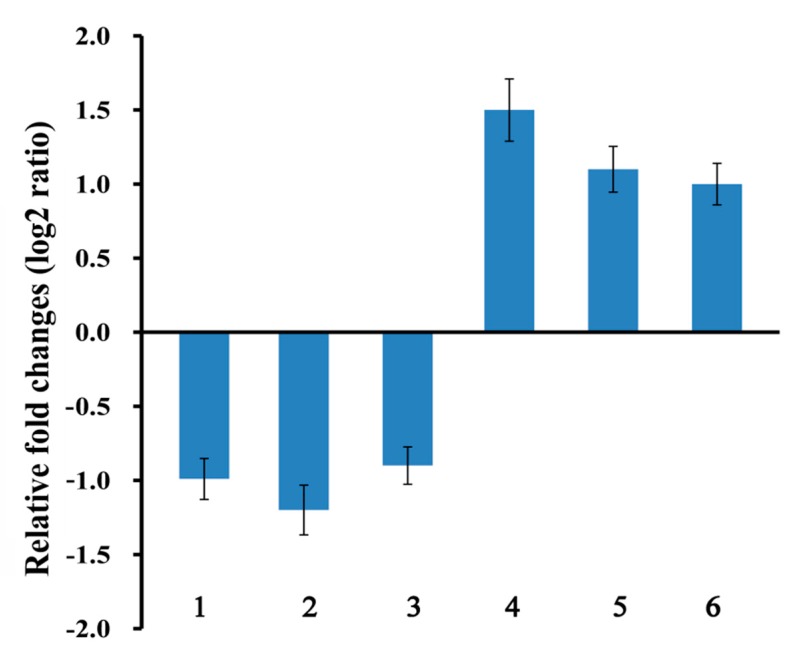
Comparison of the relative fold changes of six genes subjected to quantitative real-time polymerase chain reaction. Numbers 1, 2, 3, 4, 5, and 6 represent MC1005273, MC1004502, MC1000699, MC1004145, MC1000224, and MC1000519, respectively.

qRT-PCR analysis was performed to validate the data. Six genes (three upregulated genes including molecular function MC1004145, MC1000224, and MC1000519, and three downregulated genes including cell part (GO:0044464) MC1005273, MC1004502, and MC1000699). All the test genes were involved in the cellular regulation of biological process. In this study, given the variation in the particular values of relative fold change, the data obtained from qRT-PCR were consistent with those obtained from RNA-Seq (Figure 8).

## 4. Discussion

Salt-tolerant bacteria use many strategies to improve host plant adaptation to saline conditions. Although these mechanisms have been comprehensively investigated for decades, only a few studies have illustrated the changes in gene levels during bacterial growth. In this work, we analyzed the entire transcriptome and differential gene expression of *Halomonas* sp. MC1 after exposure to NaCl stress under normal culture conditions (Figure 1a). We observed significant changes in the expression levels of 893 genes after NaCl treatment. A total of 401 and 492 genes were upregulated and downregulated in MC1 (1.71 M NaCl), respectively (Figure 3). Notably, GO and KEGG analyses of DEGs revealed significant variations, indicating that NaCl stress considerably affected the growth of the MC1 strain. Our results also showed that the MC1 strain regulated various pathways to cope with salt stress. Studies have illustrated that bacteria can induce plants to adapt to salt stress [33,34]. Our data indicate that the bacterium itself induced its own capability to avoid hostile environments.

The total gene expression significantly changed after salt treatment (Figure 3). This result suggests that MC1 responded to abiotic stress to retain stable growth. The number of DEGs identified in this study was lower than that identified in a previous work [35]. The cellular components with the highest enrichment included the outer membrane-bounded periplasmic space (GO:0030288), plasma membrane proton-transporting ATP synthase complex (GO:0045260), and proton-transporting ATP synthase complex (GO:0045259) (Table 2). It has been reported that ATPases is involved in ATP biosynthesis and is dependent on the proton motive force created by the action of H^+^ ATPases. This enzyme is also found important in salt tolerance related genes in tomato or maize seedlings [36,37]. The number of genes in the outer membrane-bounded periplasmic space was higher than that in the control. A previous study has reported that numerous proteinslocated in the outer membrane-bounded periplasmic space help bacteria adapt to adverse conditions [38]. Our findings suggest that the MC1 strain upregulated a large number of genes involved in the membrane-bounded space to resist salt stress. A recent study identified the outer membrane-bounded periplasmic space as one of the top three enriched terms in the cell component category [39].

ATPase activity coupled with the transmembrane movement of substances (GO:0042626), active transmembrane transporter activity (GO:0022804), and primary active transmembrane transporter activity (GO:0015399) were significantly enriched in the molecular function category (Figure 4). A previous work showed that *Brevibacterium* confers salt tolerance by regulating the ATPase activity [40]. In accordance with this result, our data suggest that this activity aids bacteria in surviving under salt stress. ATPase activity is a key indicator in cell analysis [41]. Na^+^/K^+^-ATPase activities differ in bacteria exposed to different salinities [42]. Our study indicates that the high capacity of MC1 in salt tolerance was correlated with high ATPase activities.

Catabolic process (GO:0009056), proton transmembrane transport (GO:1902600), and ion transmembrane transport (GO:0034220) were significantly enriched in the biological process category. The most regulated included those involved in the catabolic process and ion transmembrane transport (Table 2). A previous study has shown that soil type substantially affects plant colonization and catabolic gene expression under the inoculation of bacterial strains [43]. Catabolic processes are crucial for the bacterial resistance to different abiotic stresses [44]. Bacterial catabolic genes are vital not only in agricultural soils, but also in marine conditions [45,46]. We also noted that ion transmembrane transport genes were regulated, suggesting that salt stimulates ion transport in the membrane space. The MC1 strain regulated genes to resist salt stress to balance the osmotic pressure. A previous study has demonstrated that salt stress induces changes including ion diffusion in the exoproteome of halotolerant bacteria [47].

The KEGG pathway database contains information on molecular interactions in known metabolic and regulatory pathways [48]. In this study, the DEGs in MC1 were matched to 104 different KEGG pathways. This result suggests that MC1 maintained survival under salt stress by regulating cellular and metabolic processes as well as pyruvate metabolism pathways such as organic and carboxylic acid catabolism. Our data showed that the most significant pathways included ABC transporters. A total of 224 genes including 129 DEGs were annotated to this pathway (Figure 5, Table 3). ABC transporters play important functions such as the high-affinity uptake of nutrients and export of cytotoxic compounds in bacterial cells [49]. We speculated that MC1 regulated ABC transporters to adapt to high salt stress. A total of 66 genes in MC1 strain were allocated to the valine, leucine, and isoleucine degradation pathway (Table 3). This pathway is highly related to residual feed intake [50]. A previous researcher has reported similar results and pointed out that leucine and isoleucine degradation occurs when *Staphylococcus aureus* is subjected to methicillin treatment in the presence of glucose [51]. Our data suggest that *Halomonas* sp. MC1 survives under salt stress by regulating the valine, leucine, and isoleucine degradation pathway. KEGG analysis yielded fewer enriched pathways than the GO analysis. The high enrichment of the outer membrane-bounded periplasmic space in the GO analysis suggests that MC1 regulates the outer membrane genes to confront salt stress. A study has reported that the membrane lipid bilayer is a regulated barrier for coping with detrimental ionic conditions [52]. We believe that our results are potentially helpful for analyzing other salt-tolerant bacteria and provide new conclusions in the analysis of gene expression under saline stress.

An enhanced understanding of a beneficial EB interaction with salt stress can improve the handling and application of plant growth-promoting microbes. Although microbial-mediated drought or heavy metal tolerance in plants [53,54] has been elucidated, our understanding of the salt tolerance mechanisms of EB remains lacking. A recent valuable work has proven that ion accumulation and the expression of ion homeostasis-related genes contribute to the NaCl-stimulated growth enhancement in halophytes, especially the common ice plant [18]. Nevertheless, the current study of *Halomonas* sp. bacteria indicates that future research features considerable potential to provide new insights into salt resistance. Collectively, our results suggest that MC1 regulates cellular and metabolic processes as well as pyruvate metabolism pathways such as organic and carboxylic acid catabolic activities to survive under salt stress. This behavior indicates that MC1 possesses a strong capability to adapt to extremely adverse environments. Given our results and the widely reported bacterial salt resistance, we highlight the mechanism of *Halomonas* sp. MC1 to elucidate the dynamics of complex salt–microbe interactions. Meanwhile, our genome-wide survey of MC1 provides candidate genes for further application of this strain.

## 5. Conclusions

We examined the effect of salt stress on the growth of *Halomonas* sp. strain MC1 by using a dataset generated through de novo assembly of next-generation sequencing data. Salt treatment caused significant changes in 893 genes including 401 upregulated and 492 downregulated genes. Our results suggest that MC1 survives under salt stress through various pathways, that is, by regulating the genes involved in the outer membrane-bounded periplasmic space, plasma membrane proton-transporting ATP synthase complex, and ABC transporters. MC1 maintained survival under salt stress by regulating cellular and metabolic processes as well as pyruvate metabolism pathways such as organic and carboxylic acid catabolic activities. Our data on DEGs can also provide potential candidate genes for the functional analyses of MC1 for survival in salt stress. Exploration of salt-resistant genes can determine which ones can be cloned or stimulated before using the bacterial strain.

## Figures and Tables

**Table 1:** Summary of the transcriptome assembly of strain MC1. Control (CK 0M) and salt-treated (1.7 M) raw read filters.

Sample	Raw Reads	Raw Bases	%≥Q30	Clean Reads	Clean Bases	Mapped-Reads	Mapped Rate (%)	GC(%)	%≥Q30	Error(%)
MC1-CK-1	13,471,728	2,020,759,200	91.21	11,691,054	1,726,157,998	10,887,922	93.13	56.93	93.90	0.0137
MC1-CK-2	15,215,384	2,282,307,600	91.1	12,979,384	1,912,783,427	11,812,868	91.01	56.67	93.81	0.0138
MC1-CK-3	16,388,692	2,458,303,800	91.5	14,177,492	2,094,489,027	13,124,998	92.58	56.44	94.01	0.0136
MC1-1	13,820,672	2,073,100,800	94.9	12,093,922	1,783,589,346	10,993,370	90.90	55.94	96.86	0.0109
MC1-2	16,604,690	2,490,703,500	93.72	14,554,380	2,145,906,752	12,859,768	88.36	56.12	96.07	0.0115
MC1-3	16,857,140	2,528,571,000	93.64	14,246,340	2,090,108,492	12,558,154	88.15	55.82	96.07	0.0115

**Table 2:** Control (MC1-CK) vs. salt-treated (MC1) GO enrichment.

Categories	Description	Ratio in Study *	Ratio in Total Genes	*p*-Value	GO ID
Cellular Component	outer membrane-bounded periplasmic space	27 (1.26%)	47 (0.87%)	0.0156	GO:0030288
	plasma membrane proton-transporting ATP synthase complex	10 (0.47%)	15 (0.28%)	0.0363	GO:0045260
	proton-transporting ATP synthase complex	10 (0.47%)	15 (0.28%)	0.0363	GO:0045259
	proton-transporting two-sector ATPase complex	10 (0.47%)	15 (0.28%)	0.0363	GO:0016469
	cytochrome o ubiquinol oxidase complex	8 (0.37%)	8 (0.15%)	0.0006	GO:0009319
	proton-transporting ATP synthase complex, catalytic core F(1)	8 (0.37%)	11 (0.20%)	0.0307	GO:0045261
	plasma membrane proton-transporting ATP synthase complex, catalytic core F(1)	8 (0.37%)	11 (0.20%)	0.0307	GO:0045262
	proton-transporting two-sector ATPase complex, catalytic domain	8 (0.37%)	11 (0.20%)	0.0307	GO:0033178
	cytochrome complex	8 (0.37%)	10 (0.18%)	0.0177	GO:0070069
Molecular Function	ATPase activity, coupled to transmembrane movement of substances	110 (5.14%)	236 (4.36%)	0.0245	GO:0042626
	active transmembrane transporter activity	73 (3.41%)	154 (2.84%)	0.0447	GO:0022804
	primary active transmembrane transporter activity	65 (3.04%)	132 (2.44%)	0.0239	GO:0015399
	ion transmembrane transporter activity	62 (2.90%)	129 (2.38%)	0.0452	GO:0015075
	ATPase coupled ion transmembrane transporter activity	26 (1.22%)	40 (0.74%)	0.0017	GO:0042625
	active ion transmembrane transporter activity	26 (1.22%)	40 (0.74%)	0.0016	GO:0022853
	proton transmembrane transporter activity	25 (1.17%)	45 (0.83%)	0.0316	GO:0015078
	inorganic anion transmembrane transporter activity	15 (0.70%)	23 (0.42%)	0.0169	GO:0015103
	ATPase-coupled anion transmembrane transporter activity	15 (0.70%)	20 (0.37%)	0.0019	GO:0043225
	proton-transporting ATP synthase activity, rotational mechanism	10 (0.47%)	15 (0.28%)	0.0363	GO:0046933
Biological Process	catabolic process	254 (11.87%)	584 (10.78%)	0.0392	GO:0009056
	proton transmembrane transport	66 (3.09%)	111 (2.05%)	0.0000	GO:1902600
	ion transmembrane transport	64 (2.99%)	134 (2.47%)	0.0492	GO:0034220
	carboxylic acid catabolic process	56 (2.62%)	99 (1.83%)	0.0005	GO:0046395
	organic acid catabolic process	56 (2.62%)	99 (1.83%)	0.0005	GO:0016054
	sulfur compound metabolic process	51 (2.38%)	901.66%)	0.0010	GO:0006790
	anion transport	44 (2.06%)	88 (1.62%)	0.0475	GO:0006820
	cellular amino acid catabolic process	27 (1.26%)	50 (0.92%)	0.0414	GO:0009063
	cellular response to chemical stimulus	19 (0.89%)	33 (0.61%)	0.0476	GO:0070887
	response to oxygen-containing compound	18 (0.84%)	28 (0.52%)	0.0105	GO:1901700

* Ratio in study: Number of differentially expressed genes/total differentially expressed genes; Ratio in Total genes: Number of differential expressed genes/total genes.

**Table 3:** Control (MC1-CK) vs. salt-treated (MC1) pathway enrichment.

Pathway	Varied Number *	Marked Number **	*p*-Value	Pathway ID
ABC transporters	129	224	0.00001	ko02010
Valine, leucine and isoleucine degradation	46	66	0.00050	ko00280
Oxidative phosphorylation	33	52	0.00832	ko00190
Synthesis and degradation of ketone bodies	14	21	0.05648	ko00072
Starch and sucrose metabolism	20	34	0.05746	ko00500
Photosynthesis	12	17	0.05826	ko00195

* Different genes. ** Total number of marked genes. The table below shows the results of the enrichment analysis of the KEGG pathway function.

## Supplementary Materials

The following are available online at https://www.mdpi.com/2076-2607/8/1/88/s1, Table S1: Oligonucleotide primers for qRT-PCR for differentially expressed gene validation.

## Abbreviations

EB	endophytic bacteria
CFU	colony-forming units
KEGG	Kyoto Encyclopedia of Genes and Genomes
GO	Gene Ontology
DEGs	differential expression genes
FDR	false discovery rate
