# Supplementary material for: Transcriptome Analysis of Ice Plant Growth-Promoting Endophytic Bacterium Halomonas sp. Strain MC1 to Identify the Genes Involved in Salt Tolerance
Source: Microorganisms. 2020 Jan 9;8(1):88. doi: 10.3390/microorganisms8010088 (PMC7022971; doi:10.3390/microorganisms8010088)
Supplement: Supplementary file 1 [file microorganisms-08-00088-s001.zip › Table S1.docx]

**Table S1** Oligonucleotide primers for qRT-PCR for differentially expressed genes validation.

| Up /down | Gene name | Forward primer (5’-3’) | Reverse primer (5’-3’) |
| --- | --- | --- | --- |
| Up regulated | MC1004145 | 5'-AGCGCGATGGACAACATGGA-3' | 5'-CTATTTGCCGTATTGCTTGCCT-3' |
|  | MC1000224 | 5’-ACACCCGCTTGACTTGATGG-3’ | 5'-GCTCCCTTTTGTAATCATCGC-3' |
|  | MC1000519 | 5'-GTGAAGAGCGGCGAGTGATA-3' | 5'-TCCTCAAGAACGATAGCCGC-3' |
| Down regulated | MC1005273 | 5'-CTTTCACGAGTGGCAGCAAG-3' | 5'-TGAGCATGGGAGATCGTGTG-3' |
|  | MC1004502 | 5'-ACCTAAGCGGGATACGTTGC-3' | 5'-TGCCATTCGTGAAAACGCTC-3' |
|  | MC1000699 | 5'-CGTCCTGATTGAGCGTAGCG-3' | 5'-GCTCAATCAGGACGCCCATG-3' |
